# Lichenase and Cellobiohydrolase Activities of a Novel Bi-Functional β-Glucanase from the Marine Bacterium *Streptomyces* sp. J103

**DOI:** 10.3390/md22120558

**Published:** 2024-12-13

**Authors:** Youngdeuk Lee, Eunyoung Jo, Yeon-Ju Lee, Min Jin Kim, Navindu Dinara Gajanayaka, Mahanama De Zoysa, Gun-Hoo Park, Chulhong Oh

**Affiliations:** 1 Jeju Bio Research Center, Korea Institute of Ocean Science & Technology, Jeju-si 63349, Republic of Korea; lyd1981@kiost.ac.kr (Y.L.); jey8574@kiost.ac.kr (E.J.); navindu@kiost.ac.kr (N.D.G.); gunhoopark@kiost.ac.kr (G.-H.P.); 2 Marine Biotechnology & Bioresource Research Department, Korea Institute of Ocean Science & Technology, Busan 49111, Republic of Korea; yjlee@kiost.ac.kr (Y.-J.L.); minjin@kiost.ac.kr (M.J.K.); 3 Department of Marine Biotechnology, KIOST School, Korea National University of Science and Technology, Daejeon 34113, Republic of Korea; 4 College of Veterinary Medicine and Research Institute of Veterinary Medicine, Chungnam National University, Daejeon 34134, Republic of Korea; mahanama@cnu.ac.kr

**Keywords:** lichenase, β-1,3-1,4-glucanase, exo-β-1,4-glucanase, cellobiohydrolase, *Streptomyces* sp. J103

## Abstract

In this study, we report the molecular and enzymatic characterisation of Spg103, a novel bifunctional β-glucanase from the marine bacterium *Streptomyces* sp. J103. Recombinant Spg103 (rSpg103) functioned optimally at 60 °C and pH 6. Notably, Spg103 exhibited distinct stability properties, with increased activity in the presence of Na+ and EDTA. Spg103 displays both lichenase and cellobiohydrolase activity. Despite possessing a GH5 cellulase domain, FN3 and CBM3 domains characteristic of cellulases and CBHs, biochemical assays showed that rSpg103 exhibited higher activity towards mixed β-1,3-1,4-glucan such as barley β-glucan and lichenan than towards beta-1,4-linkages. The endolytic activity of the enzyme was confirmed by TLC and UPLC-MS analyses, which identified cellotriose as the main hydrolysis product. In addition, Spg103 exhibited an exo-type activity, selectively releasing cellobiose units from cellooligosaccharides, which is characteristic of cellobiohydrolases. These results demonstrate the potential of Spg103 for a variety of biotechnological applications, particularly those requiring tailor-made enzymatic degradation of mixed-linked β-glucans. This study provides a basis for further structural and functional investigations of the bifunctional enzyme and highlights Spg103 as a promising candidate for industrial applications.

## 1. Introduction

Among the various types of β-glucans, β-1,3-1,4-glucans, predominantly found in cereals, such as barley, are distinguished by their unique structure, which alternates between β-1,3 and β-1,4 glycosidic linkages [1]. Lichenan, a similar polysaccharide from lichen, shares structural characteristics with β-1,3-1,4-glucans, but differs in its specific arrangement and biological origin. Similarly to β-glucans, lichenan can be enzymatically hydrolysed into glucose through enzymes that specifically target its β-1,3 and β-1,4 linkages [2].

Endo β-glucanases (EC 3.2.1.4) randomly hydrolyse accessible intramolecular β-1,4-glucosidic bonds within cellulose chains, producing new chain ends [3]. Exoglucanases (EC 3.2.1.91)/cellobiohydrolase (CBH) cleave cellulose chains from the ends to release soluble cellobiose or glucose, while β-glucosidases further hydrolyse cellobiose to glucose [4].

Mixed-linkage β-1,3-1,4-glucans are linear polymers composed of up to 1200 β-glucosyl units, where segments of three to four β-1,4 linkages are interspersed with single β-1,3 linkages [2,5]. β-1,3-1,4-glucanase (EC 3.2.1.73, commonly referred to as β-glucanase or lichenase) is responsible for cleaving the β-1,4-glycosidic bonds adjacent to β-1,3-glycosidic linkages in these mixed-linkage glucans and lichenan. However, this enzyme cannot cleave the β-1,4-glycosidic bonds found in carboxymethyl cellulose (CMC).

Both β-glucans and lichenan are recognised as important bioactive compounds due to their various health benefits. Their potential application in nutrition and biotechnology has attracted significant scientific interest. As a dietary fibre, glucans play essential roles in promoting gut health, modulating the immune system, and regulating blood glucose and cholesterol levels [6,7]. Their unique structure, which combines β-1,3 and β-1,4 linkages, requires specific enzymatic activity for effective degradation.

Most known lichenases originate from bacteria and are classified within the glycoside hydrolase family 16 [8,9]. The most extensively studied lichenases are produced by *Bacillus* species, whereas plant enzymes with similar specificities are grouped under family 17 [8,10]. To date, only two bacterial species, *Bacillus subtilis* A8-8 and *Ruminococcus albus* 8, have been reported to produce GH5 family lichenases, excluding those from uncultured bacteria, according to the CAZy database. In this study, we identified a novel lichenase from the marine bacterium *Streptomyces* sp. J103, which contains a cellulase domain classified within the glycoside hydrolase family 5 and analysed its biochemical properties and mode of action.

## 2. Results

### 2.1. Molecular Characteristics of Spg103

**Figure 1:**
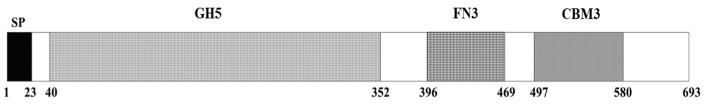
Functional domain structure of Spg103. SP: signal peptide, GH5: glycosyl hydrolase family 5 (cellulase domain), FN3: fibronectin type 3 domain, CBM3: carbohydrate-binding module 3 domain.

The *spg103* gene has a 1932 bp open reading frame, encoding 643 amino acids with a molecular weight of 67.5 kDa. The N-terminal region of the sequence contains a Spg103 signal peptide with 23 amino acids for extracellular protein secretion. The signal peptide is followed by a cellulase domain (a catalytic domain) from glycosyl hydrolase family 5 (40–352). The fibronectin type 3 domain (FN3, 396–469) and the carbohydrate-binding module 3 domain (497–580) were found in the C-terminal region (Figure 1).

Spg103 showed the highest amino acid similarity with hypothetical and uncharacterised protein sequences obtained from genome sequencing without biochemical characterisation. The CAZY database contains only three characterised lichenases from two bacterial species, *Bacillus subtilis* A8-8 and *Ruminococcus albus* 8, although three lichenases have been identified in uncultured bacteria [11,12,13]. Compared to biochemically characterised GH5 family cellulases and lichenases, *spg103* showed the highest identity and similarity with lichenases from *B. subtilis* A8-8. The identity and similarity were 26.6 and 39.6%, respectively. Compared to the amino acid sequence of characterised GH16 lichenase from bacteria in the CAZY database, very low identities (approximately 10%) and similarities (less than 20%) were observed (Table 1).

### 2.2. Substrate Specificities

**Figure 2:**
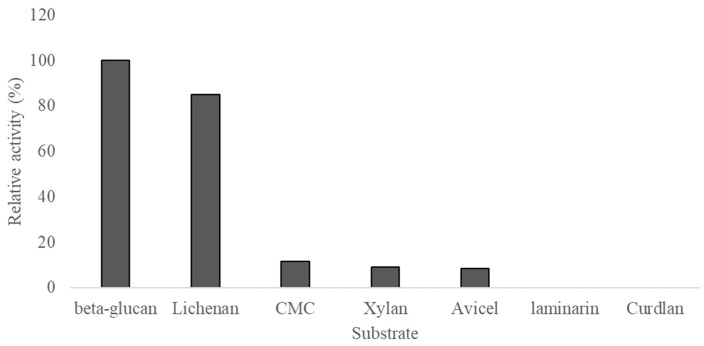
The relative substrate-specific activity of rSpg103.

Substrate specificity screening showed that rSpg103 displayed high activity against barley beta-glucan and lichenan. The relative substrate-specific activity is shown in Figure 2. Compared to barley beta-glucan, which showed the highest activity, lichenan displayed a relatively high activity of 85%. In contrast, CMC, xylan, and avicel exhibited very low relative activities (11.6, 9.1, and 8.4%, respectively). Meanwhile, no activity was observed against laminarin and cudlan, which are composed solely of β-1,3 linkages.

### 2.3. Biochemical Properties of rSpg103

**Figure 3:**
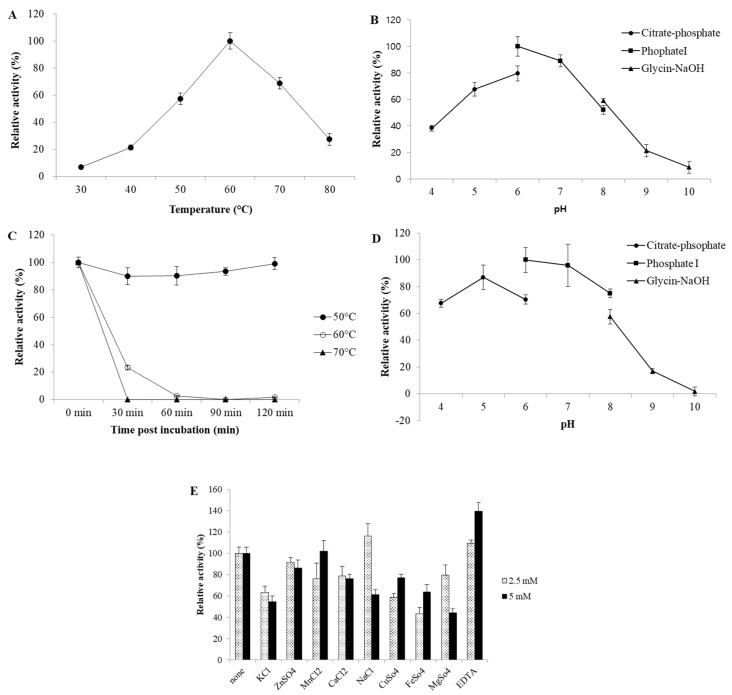
The biochemical properties of rSpg103. (**A**) Optimal temperature was determined based on activity at various temperatures (30–80 °C). (**B**) Optimal pH of rSpg103 was determined by measuring the activity in the citrate-phosphate buffer (pH 4.0–6.0), phosphate buffer (pH 6.0–8.0), glycine-NaOH (pH 8.0–9.0). (**C**) Thermostability of rSpg103. Residual activity was determined after pre-incubation at 50, 60, and 70 °C. (**D**) pH stability of rSpg103. Residual activity was measured at 60 °C for 10 min after the enzyme was incubated at pH 4.0–10.0 with the above buffers for 24 h at 4 °C. (**E**) Metal ion effects of rSpg103 were determined using various metal ions and chelator. The experiments were performed in triplicate. Error bars represent mean ± SD.

To determine the biochemical properties of rSpg103, enzyme assays were performed to determine the optimal temperature, pH, thermostability, and metal and chelating ion effects. The optimal temperature was 60 °C (Figure 3A). In the thermostability assay, rSpg103 was highly stable at 50 °C for 120 min, maintaining its initial activity throughout the duration of the assay (Figure 3C). Meanwhile, rSpg103 lost all its activity within 30 min at 70 °C. Similarly, it showed only approximately 20% of its activity after pre-incubation for 30 min at 60 °C, and no activity was observed after 60 min of pre-incubation. rSpg103 exhibits a melting temperature (T_m_) of 68.4 °C, indicating its structural stability. However, its aggregation temperature (T_agg_) was observed to be 61.2 °C. The optimum pH was pH 6 (Figure 3B) and more than 80% of its activity was retained at pH 7.0. rSpg103 exhibited optimal pH stability at pH 6.0–7.0 and retained more than 60% of the initial activities after incubation (Figure 3D).

The enzyme activity of rSpg103 was not or poorly affected by K^+^, Zn^2+^, Mn^2+^, Ca^2+^, Cu^2+^, Fe^3+^, and Mg^2+^ ions, but its activity increased by approximately 16% in the presence of Na^+^ ions. Interestingly, rSpg103 showed increased activity in the presence of 2.5 and 5 mM EDTA (Figure 3E). The V_max_ and K_m_ values were 47.9 U/mg and 1.3 mg/mL for barley beta-glucan, and 43.6 U/mg and 1.5 mg/mL for lichenan, respectively.

### 2.4. TLC

**Figure 4:**
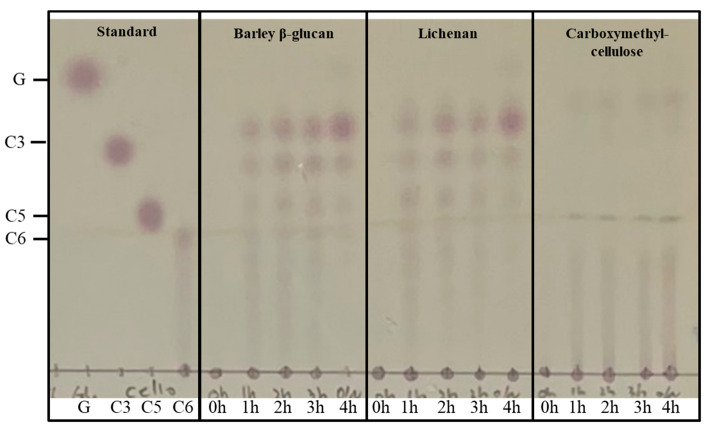
Thin layer chromatography of the time course hydrolytic products of rSpg103 towards barley beta-glucan, lichenan, and CMC. G: d-glucose, C3: cellotriose, C5: cellopentaose, C6, cellohexaose.

To determine the mode of action of rSpg103, lichenan, and β-glucan were hydrolysed, and the products were analysed via TLC. A time course degradation of both β-glucan and lichenan showed various depolymerised oligosaccharides at the beginning (Figure 4). As the reaction time progressed, the high molecular weight products gradually decreased, and the degree of polymerisation (DP) 3 was detected as the main product after 120 min. Interestingly, the hydrolysis product of CMC with rSpg103 did not contain DP3, DP4, or DP5. However, a faint spot predicted to be DP2 was observed.

**Figure 5:**
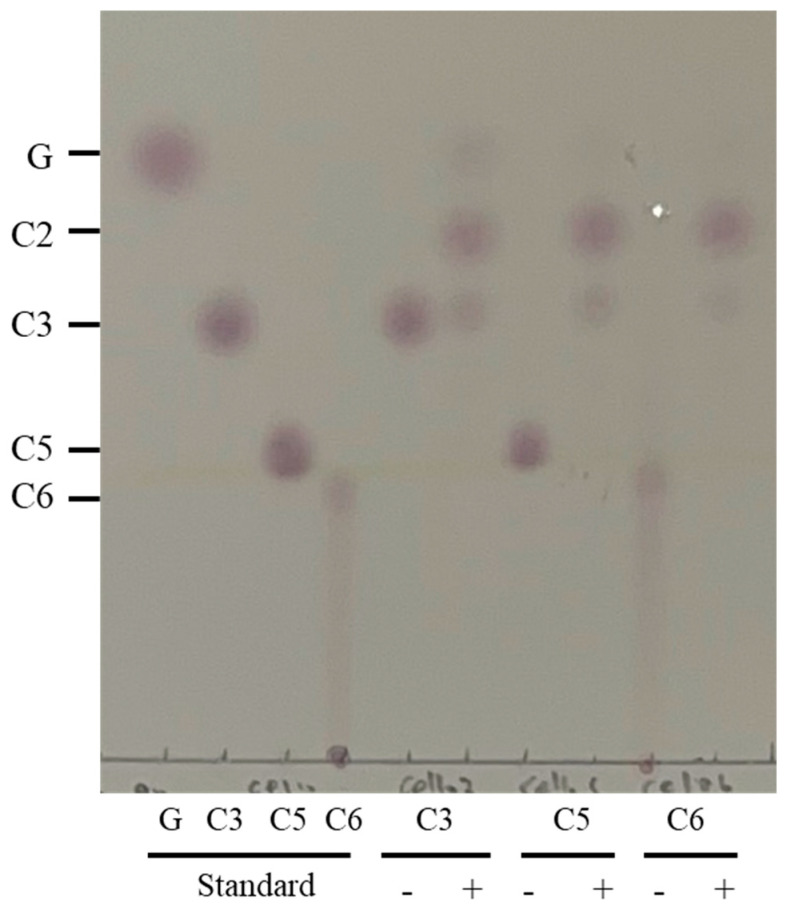
Thin layer chromatography of rSpg103 towards cello-oligosaccharides. G: D-glucose, C3: cellotriose, C5, cellopentaose, C6: cellohexaose. The “+” and “−” symbols represent the presence or absence of the rSpg103 enzyme, respectively.

To further analyse the cellulolytic mode of action of rSpg103, cellobiose, cellotriose, and cellopentaose were hydrolysed using rSpg103 (Figure 5). Recombinant Spg103 degraded cellotriose, cellopentaose, and cellohexaose. rSpg103 degraded cellotriose into cellobiose and d-glucose, cellopentose into cellotriose and cellobiose, and cellohexose into cellobiose.

### 2.5. Hydrolytic Products of rSpg103

**Figure 6:**
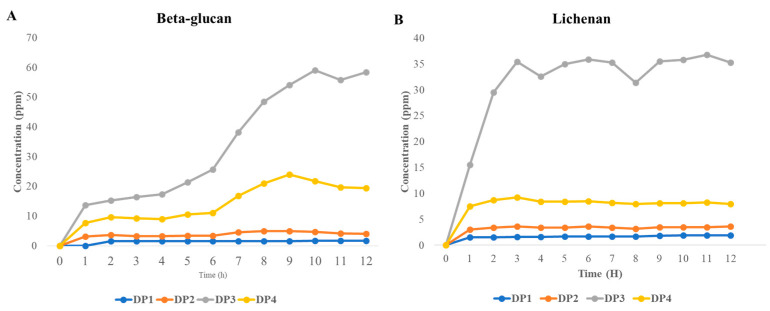
The UPLC-MS results of beta-glucan (**A**) and lichenan (**B**) hydrolysed by rSpg103. DP: degree of polymerization.

The results of the UPLC analysis for the hydrolysed products of barley beta-glucan and lichenan are presented in Figure 6. The highest concentration of beta-glucan hydrolysis products was observed (58.3 ppm), with cellotriose identified as the main product. The second highest concentration (19.4 ppm) was attributed to cellotetraose. Cellobiose and D-glucose were observed 2 h post-reaction at 3.6 and 1.5 ppm, respectively. Although the quantity of the products was minimal, a slight increase was observed with time until 12 h, when they reached 4 and 1.7 ppm, respectively. In the hydrolysis of lichenan, as with beta-glucan, the main product 12 h after the reaction was cellotriose (35.3 ppm), followed by cellotetraose at 8 ppm. The concentrations of cellobiose and D-glucose were 3.6 and 1.9 ppm, respectively. As observed with beta-glucan, the concentrations exhibited a slight increase over time. In both beta-glucan and lichenan, cellotriose was the main product, exhibiting a rapid increase in concentration. Cellotetraose had the second highest concentration in both substrates, whereas cellobiose and D-glucose were observed at lower concentrations and increased at a slower rate.

## 3. Discussion

In the present study, we investigated the molecular characteristics and biochemical properties of a novel lichenase isolated from *Streptomyces* spp. J103. This is the first report of a cellulase domain-containing bifunctional lichenase isolated from *Streptomyces*.

We identified a predicted β-glucanase from *Streptomyces* by analysing the whole genome sequence of *Streptomyces* sp. J103. The amino acid sequence of the predicted β-glucanase, Spg103, showed the highest identity and similarity with the GH5 cellulase domain containing hypothetical proteins. Therefore, a pairwise alignment analysis was performed using biochemically characterised GH5 family cellulase domain-containing proteins, including cellulases and lichenases. In pairwise alignment results, Spg103 showed the highest identity (26.6%) and similarity (39.6%) with endo-acting lichenases from *Bacillus subtilis* A8-8 compared to GH5 family cellulases and lichenases. Spg103 showed very low amino acid identity and similarity to other characterised lichenases. The observed low identity and similarity may be attributed to the limited data available on GH5 family lichenases. Only five lichenases belonging to the GH5 family have been reported to date, which limits the number of lichenase sequences available for amino acid sequence characterisation.

Amino acid sequence analysis revealed that the Spg103 protein consisted of a signal peptide and GH5 family cellulase domain at the N-terminus, FN3 domain in the middle, and CBM3 at the C-terminus. FN3 is a domain often found in cellobiohydrolases [14], and CBM3 has been reported to have a cellulose-binding function. This functional domain structure of Spg103 showed more similarity to extracellular exo-beta-1,4-glucanase, but interestingly, it exhibited higher activity on mixed beta-glucan.

The substrate specificity screening assay revealed higher activity mixed-linkages glucans in rSpg103, such as barely β-glucan and lichenan, compared to birchwood xylan, avicel, and carboxymethyl-cellulose. rSpg103 could hydrolyse the β-1,3-linkages present in β-1,3-1,4-glucans, but exhibited no activity on β-1,3-glucnas, such as laminarin. This result was similar to the previously reported GH5 family containing lichenases. The Ra0453 protein from the rumen bacterium *Ruminococcus albus* 8 demonstrated very weak enzymatic activity towards avicel and CMC, which only comprise β-1,4-linkages, but it showed 1000 times higher activity against lichenan, a substrate with interlaced β-1,3 and β-1,4-linkages [15]. Cel5A derived from the metagenome of uncultured microorganisms was also highly active against mixed beta-glucan such as lichenan and barley β-glucan, but there was no activity against beta-1,4-glucans such as crystal-formed cellulose and Avicel. Cel5A was also unable to hydrolyse beta-1,3-linked glucan [13].

It has been reported that CBMs enhance the activity of the enzymes on insoluble substrates by increasing the effective enzyme concentration on the polysaccharide surface [15]. Salmeán et al. [16] have reported that mixed-linkage β-1,3-1,4-glucans (MLG) is a common constituent of brown algal cell walls. In contrast to the soluble terrestrial beta-glucan, the brown algae MLGs are insoluble. It may be posited that the CBM3 domain of Spg103 plays an important role in the utilisation of insoluble MLGs in brown algae.

Substrate specificity assays suggested that Spg103 is an endo-acting lichenase. Therefore, the biochemical properties towards β-glucan and lichenan were analysed. Previously reported lichenases have shown optimal temperatures ranging from 40 to 60 °C, and optimal pH has been observed at a pH range of 5.5–8.0 [17,18]. Similarly, rSpg103 also showed optimal temperature and pH at 60 °C and pH 6.0, respectively. Most enzymes reported to date are inhibited by EDTA [19]. However, interestingly, rSpg103 activity was enhanced by 40% in the presence of EDTA. Similar results have also been reported previously. Naika et al. [20] reported that the enzymatic activity of the endoglucanase identified in *Aspergillus aculeatus* was enhanced by 1.5-fold in the presence of 2 mM EDTA, compared to that of the control. The thermal stability results showed that rSpg103 was stable at 50 °C, but showed a significant decrease at temperatures above 60 °C. The T_agg_ value of rSpg103 was found to be lower than the T_m_, indicating that aggregation occurs prior to the complete unfolding of rSpg103, therefore a rapid decrease in enzymatic activity of rSpg103 above 60 °C is likely due to the formation of aggregates that impair its functional conformation.

In time course TLC analysis, rSpg103 hydrolysed lichenan and β-glucan, generating an intermediate product larger than cellotetraose in the early stage of the reaction. UPLC-MS analysis showed that rSpg103 hydrolysed lichenan and beta-glucan, rapidly generating DP3 and DP4. This suggests that rSpg103 displays the typical features of an endo-acting enzyme. Additionally, we attempted to hydrolyse cello-oligosaccharides, including cellotriose, cellopentaose, and cellohexaose. We found that all of them could be hydrolysed to produce cellobiose as the main product. The production of cellobiose from cello-oligosaccharides is only possible if beta-1,4-bonds can be cleaved. However, in the substrate screening assay, rSpg103 showed very low activity against CMC, which is composed only of beta-1,4-bonds. The UPLC-MS results showed that the disaccharide and monosaccharide concentrations increased very slowly. This appears to be a separate reaction from the endo-activity, which rapidly cleaved triose and tetraose. Therefore, we predicted that rSpg103 would exhibit exo-activity against beta-1,4-bonds. To verify the hypothesis that rSpg103 exerts endo- and exo-activities against mixed beta-glucans and beta-1,4-glucans, respectively, we attempted to hydrolyse CMC with rSpg103 and confirm its hydrolysis pattern using TLC. RSpg103 degraded CMC into cellobiose without any intermediate products. The TLC and ULPC-MS results substantiated the hypothesis that rSpg103 can recognise the beta-1,4-bond in polysaccharides exclusively and subsequently degrade them into disaccharides. This is a distinctive property not observed in typical lichenases, which demonstrate specific cleavage of beta-1,4-linkages in proximity to beta-1,3-linkages. These findings indicate that rSpg103 exhibits exo-type activity, specifically the ability to hydrolyse beta-1,4-linkages on a two-sugar basis. This feature of rSpg103 is similar to that of CBH among exo-beta-1,4-glucanases. CBH specifically breaks down cellulose by cleaving cellobiose units from the ends of cellulose chains. It acts exolytically, meaning that it works from both the reducing and non-reducing ends of the cellulose chains, progressively releasing cellobiose [21,22].

This bifunctional nature of Spg103 suggests its potential as a candidate for use in a variety of industrial applications.

## 4. Materials and Methods

### 4.1. Identification and Molecular Characterisation of spg103

We previously isolated the *Streptomyces* sp. strain J103 and analysed its genome sequence [23]. A unique nucleotide sequence that showed the highest homology to known β-glucanases was identified using the Basic Local Alignment Search Tool (BLAST) algorithm and named Spg103. The signal peptide sequence of Spg103 was predicted using the SignalP 5.0, server (http://www.cbs.dtu.dk/services/SignalP/, accessed on 12 January 2024) [24]. The similarity and identity of the nucleotide and amino acid sequences were analysed using NCBI BLAST and EMBL-EBI pairwise alignment tools (http://www.ebi.ac.uk/Tools/psa/, accessed on 12 January 2024). The Simple Modular Architecture Research Tool (SMART; http://smart.embl-heidelberg.de/, accessed on 12 January 2024) was used to identify the functional domains of Spg103. The amino acid sequence of Spg103 was submitted to NCBI GenBank under accession number PQ582394.

### 4.2. Cloning of the spg103 Gene

Primers were designed to amplify the *spg103* gene as follows: forward, 5′-gag agg atc cGC GGG TAC GGC GGG AGC G-3′, sense; and reverse, 5′-gag aaa gct ttc aGG GTT CGA TGC CGT TCG CGA GG-3′, antisense. The restriction sites for *BamH*I and *Hind*III are highlighted. The *spg103* gene was amplified via PCR using genomic DN A isolated from *Streptomyces* sp. J103 as a template. The amplified products were purified using a PCR Purification Kit (Bioneer, Daejeon, Republic of Korea). The purified PCR product and pMal-c2x expression vector (New England Biolabs, Ipswich, MA, USA) were digested with *BamH*I and *Hind*III restriction enzymes and then purified using a gel purification kit (Bioneer, Daejeon, Republic of Korea). The gel-purified PCR products were ligated into the digested pMal-c2x vector. The ligated DNA was transformed into *Escherichia coli* DH5α. Then, spg103-pMal-c2x fusion plasmid was purified using AccuPrep^®^ Nano-Plus Plasmid Mini Extraction kit (Bionner, Daejeon, Republic of Korea), and the clones were again transformed into *E. coli* BL21 (DE3) expression cells.

### 4.3. Overexpression and Purification of Recombinant Spg103 (rSpg103)

*E. coli* cells harbouring the spg103-pMal-c2x fusion plasmid were inoculated in 5 mL of Luria broth containing ampicillin (final concentration 100 µg/mL) and incubated at 37 °C overnight. The sub-culture was re-inoculated into 250 mL of fresh rich broth (10 g trypton, 5 g yeast extract, 5 g NaCl, and 2 g glucose per 1 L) containing 100 mg/mL of ampicillin and incubated until a mid-logarithmic phase (OD_600nm_ = 0.6–0.7). To overexpress the recombinant protein, isopropyl-β-d-thiolgalactoside (IPTG) was added to a final concentration of 0.05 mM. The cultures with IPTG were incubated at 20 °C for 20 h. The cells were harvested via centrifugation at 1800× *g* for 15 min. The collected cells were re-suspended in 25 mL of column buffer (20 mL of 1 M Tris-HCl pH 7.4 and 11.7 g of NaCl per 1 L) and frozen at −20 °C overnight.

The frozen cells were thawed on ice and then disrupted using sonication. The supernatants were separated via centrifugation at 13,000× *g* for 20 min at 4 °C. rSpg103 was purified from the soluble fraction using the pMal^TM^ Protein Fusion and Purification System (New England Biolabs, Ipswich, MA, USA) and analysed using SDS-PAGE. The concentration of the purified recombinant proteins was determined using a BCA Protein Assay Reagent Kit (Thermo Fisher Scientific Inc., Waltham, MA, USA).

### 4.4. Enzyme Activity Assay

To investigate substrate specificity, the activities of rSpg103 against Avicel (Sigma-Aldrich, St. Louis, MO, USA), CMC (Sigma-Aldrich, St. Louis, MO, USA), barley beta-glucan (Megazyme, Bray, Co., Wicklow, Ireland), lichenan from Icelandic moss (Megazyme, Bray, Co. Wicklow, Ireland), and birchwood xylan (Megazyme, Bray, Co., Wicklow, Ireland) were determined. Enzyme activities were measured as the reduced sugar release in the hydrolytic reaction. The reduced sugar content was determined using a modified 3,5-dinitrosalicylic acid method [25]. The assay was carried out at 60 °C for 10 min in 200 μL containing 10 μL of diluted enzyme solution, 100 μL of 2% substrate, and 90 μL of buffer. One activity unit was defined as the amount of enzyme needed to release 1 μmol reducing sugar per 1 min (using glucose as the reference). An optimal temperature assay was carried out at 30–80 °C with 10 °C intervals. The optimal pH was measured at pH 4.0–10.0 with intervals of pH 1.0. To analyse thermostability, rSpg103 was pre-incubated at temperatures of 50, 60, and 70 °C, and enzyme activities were measured every 30 min until 120 min. The relative activity was calculated based on a maximum activity of 100%. Additionally, T_m_ and T_agg_ were measured and calculated using the UNCLE protein stability screening platform (Unchained Laboratories, Pleasanton, CA, USA). To determine the kinetic parameters of Spg103, its activities were measured at various substrate concentrations ranging from 0.05 to 1% under standard conditions. *K*_m_ and *V*_max_ were calculated using GraphPad Prism (version 8.3.1; GraphPad Software, Inc., La Jolla, CA, USA). The *K*_m_ values in mg/mL were employed due to the inherent variability in the molecular weight of the substrate, which is not constant.

### 4.5. Hydrolytic Action

Thin layer chromatography (TLC) was used to analyse the hydrolytic patterns of rSpg103. D-glucose (Sigma-Aldrich), cellotriose, and cellopentaose (all from Biosynth, Staad, Switzerland) were used as standards. The beta-glucan and lichenan were hydrolysed by rSpg103 under standard conditions. The hydrolytic products were applied to a silica gel 60 F254 (Merck, Darmstadt, Germany) developed using a solvent system of n-butanol:ethanol:dH_2_O [3:2:2 (*v/v*)]. Spots were visualised by spraying orcinol dip reagent (80 mg of orcinol monohydrate was dissolved in 160 mL of acetone, and 8 mL of sulfuric acid was added), followed by heating at 110 °C in a drying oven for 10 min.

### 4.6. Product Detection Using UPLC-MS/MS

To analyse the mode of action of rSpg103, barley beta-glucan, and lichenan were hydrolysed by rSpg103 under standard conditions for 12 h at 1 h intervals. The hydrolytic products of beta-glucan and lichenan obtained from treatment with rSpg103 were analysed using UHPLC-MS/MS. The UHPLC-MS/MS system consisted of an ACQUITY ultra-performance liquid chromatograph (Waters, Milford, MA, USA) coupled with an API 4000 triple quadrupole mass spectrometer (AB Sciex, Foster City, CA, USA) equipped with a Turbo Ion Source operating in negative mode. Chromatographic separation was conducted on an ACQUITY UPLC BEH amide column (2.1 × 100 mm, 1.7 µm particle size) equipped with an ACQUITY UPLC BEH Amide 1.7 µm Van-Guard Pre-column. The column temperature was maintained at 60 °C. Mobile phase A consisted of 10 mM ammonium acetate in H_2_O/acetonitrile (95:5, *v/v*, pH 6.82), whereas mobile phase B consisted of 10 mM ammonium acetate in acetonitrile/H_2_O (95:5, *v/v*, pH 8.22). Carbohydrates were separated using the following gradient programme at a flow rate of 0.25 mL/min over the course of 10 min: starting with a linear increase from 2% A to 40% A in 6.0 min, then maintained at 40% A for 1.6 min. The mobile phase was allowed to return to the initial condition within 0.1 min, followed by column re-equilibration for 2.3 min. The injection volume was 10 µL. The compounds of interest were detected using MS/MS in negative ion multiple reaction monitoring mode.

Turbo V ion source parameters were common for all analytes: the capillary voltage was −4500 V, and the source temperature was 550 °C. The pressure of the curtain gas (N_2_) was set at 25 psi. The pressure for the nebulization and vaporisation gases was set to 60 psi. The entrance potential and collision cell exit were −80 and −10 V, respectively. The de-clustering potential and collision energy were optimised for each analyte.

## 5. Conclusions

In the present study, Spg103 was identified from *Streptomyces* J103, and molecular characterisation and biochemical characterisation of the recombinant protein were performed. Furthermore, the degradation pattern of the recombinant protein was analysed. The recombinant enzyme showed the optimum temperature and pH at 60 °C and pH 6.0, respectively, and uniquely, the enzyme activity was enhanced by EDTA. This enzyme showed both endolytic lichenase and exolytic cellobiohydrolase activities. This is a rare example of a bifunctional enzyme, and further studies are required to explore its structural properties, engineering possibilities for functional enhancement, and mechanistic aspects to broaden its applications in various industries.

## Figures and Tables

**Table 1:** Glycosyl hydrolase family 5 domain containing beta-1,3-1,4-glucanases.

Title 1	EC. No.	Enzyme Name (Protein Name)	Similarity/Identity (%)	Accession No.
*Streptomyces* sp. J103	3.2.1.73 3.2.1.91	Lichenase (Spg103)	PQ582394	Present study
*Bacillus subtillis* A8-8	3.2.1.4	β-1,3-1,4-glucnase/cellulase (CelA)	26.6/39.6	AAO63626.1
*Ruminococcus albus* 8	3.2.1.73	Lichenase (Ra0453)	10/17.7	EGC02962.1
	3.2.1.73	Lichenase (Ra2830)	20.7/30.8	EGC04285.1
Uncultured bacterium	3.2.1.73	Exoglucanase (Exg-D)	11.5/20.5	AMO13174.1
Uncultured bacterium	3.2.1.73 3.2.1.74	Endoglucanase (Cel5A)	12.8/22.1	ABA02176.1
Uncultured bacterium	3.2.1.73	Endoglucanase (GH5-g)	11.4/18.4	AIT97140.1

## Data Availability

The original contributions presented in the study are included in the article, further inquiries can be directed to the corresponding author.

## References

[1] Mirończuk-Chodakowska I., Kujawowicz K., Witkowska A.M. (2021). Beta-Glucans from Fungi: Biological and Health-Promoting Potential in the COVID-19 Pandemic Era. Nutrients.

[2] Huang Z., Ni G.R., Wang F., Zhao X.Y., Chen Y.D., Zhang L.X., Qu M.R. (2022). Characterization of a Thermostable Lichenase from Bacillus subtilis B110 and Its Effects on ?-Glucan Hydrolysis. J. Microbiol. Biotechnol..

[3] Percival Zhang Y.H., Himmel M.E., Mielenz J.R. (2006). Outlook for cellulase improvement: Screening and selection strategies. Biotechnol. Adv..

[4] Lynd L.R., Weimer P.J., van Zyl W.H., Pretorius I.S. (2002). Microbial cellulose utilization: Fundamentals and biotechnology. Microbiol. Mol. Biol. Rev..

[5] Buckeridge M.S., Vergara C.E., Carpita N.C. (1999). The mechanism of synthesis of a mixed-linkage (1→3), (1→4)β-D-glucan in maize. Evidence for multiple sites of glucosyl transfer in the synthase complex. Plant Physiol..

[6] Singh R.P., Bhardwaj A. (2023). β-glucans: A potential source for maintaining gut microbiota and the immune system. Front. Nutr..

[7] Tiwari U., Cummins E. (2011). Meta-analysis of the effect of β-glucan intake on blood cholesterol and glucose levels. Nutrition.

[8] Grishutin S.G., Gusakov A.V., Dzedzyulya E.I., Sinitsyn A.P. (2006). A lichenase-like family 12 endo-(1→4)-β-glucanase from Aspergillus japonicus: Study of the substrate specificity and mode of action on β-glucans in comparison with other glycoside hydrolases. Carbohydr. Res..

[9] Mat Yajit N.L., Fazlin Hashim N.H., Illias R.M., Abdul Murad A.M. (2024). Expression and biochemical characterization of a novel thermostable alkaline β-1,3–1,4-glucanase (lichenase) from an alkaliphilic *Bacillus lehensis* G1. Protein Expr. Purif..

[10] Hrmova M., Fincher G.B. (2001). Structure-function relationships of β-D-glucan endo- and exohydrolases from higher plants. Plant Mol. Biol..

[11] Mafa M.S., Dirr H.W., Malgas S., Krause R.W.M., Rashamuse K., Pletschke B.I. (2020). A Novel Dimeric Exoglucanase (GH5_38): Biochemical and Structural Characterisation towards its Application in Alkyl Cellobioside Synthesis. Molecules.

[12] Mackenzie A.K., Naas A.E., Kracun S.K., Schückel J., Fangel J.U., Agger J.W., Willats W.G., Eijsink V.G., Pope P.B. (2015). A polysaccharide utilization locus from an uncultured bacteroidetes phylotype suggests ecological adaptation and substrate versatility. Appl. Environ. Microbiol..

[13] Voget S., Steele H.L., Streit W.R. (2006). Characterization of a metagenome-derived halotolerant cellulase. J. Biotechnol..

[14] Kataeva I.A., Seidel R.D., Shah A., West L.T., Li X.L., Ljungdahl L.G. (2002). The fibronectin type 3-like repeat from the *Clostridium thermocellum* cellobiohydrolase CbhA promotes hydrolysis of cellulose by modifying its surface. Appl. Environ. Microbiol..

[15] Gill J., Rixon J.E., Bolam D.N., McQueen-Mason S., Simson P.J., Williamson M.P., Hazlewood G.P., Gilbert H.J. (1999). The Type II and X cellulose-binding domains of *Pseudomonas xylanase* A potentiate catalytic activity against complex substrates by a common mechanism. Biochem. J..

[16] Salmeán A.A., Duffieux D., Harholt J., Qin F., Michel G., Czjzek M., Willats W.G.T., Hervé C. (2017). Insoluble (1 → 3), (1 → 4)-β-D-glucan is a component of cell walls in brown algae (Phaeophyceae) and is masked by alginates in tissues. Sci. Rep..

[17] Iakiviak M., Mackie R.I., Cann I.K. (2011). Functional analyses of multiple lichenin-degrading enzymes from the rumen bacterium *Ruminococcus albus* 8. Appl. Environ. Microbiol..

[18] Ohara H., Noguchi J., Karita S., Kimura T., Sakka K., Ohmiya K. (2000). Sequence of egV and properties of EgV, a *Ruminococcus albus* endoglucanase containing a dockerin domain. Biosci. Biotechnol. Biochem..

[19] Auld D.S. (1988). [11] Use of chelating agents to inhibit enzymes. Methods Enzymol..

[20] Naika G.S., Tiku P.K. (2011). Influence of ethylenediaminetetraacetic acid (EDTA) on the structural stability of endoglucanase from *Aspergillus aculeatus*. J. Agric. Food Chem..

[21] Bayer E.A., Shoham Y., Lamed R. (2006). Cellulose-decomposing bacteria and their enzyme systems. Prokaryotes.

[22] Sinha T., Sharma K., Yazdani S.S., Goyal A., Sharma K. (2023). Chapter 4—Cellobiohydrolases. Glycoside Hydrolases.

[23] Marasinghe S.D., Jo E., Hettiarachchi S.A., Lee Y., Eom T.Y., Gang Y., Kang Y.H., Oh C. (2021). Characterization of glycoside hydrolase family 11 xylanase from *Streptomyces* sp. strain J103; its synergetic effect with acetyl xylan esterase and enhancement of enzymatic hydrolysis of lignocellulosic biomass. Microb. Cell Factories.

[24] Petersen T.N., Brunak S., von Heijne G., Nielsen H. (2011). SignalP 4.0: Discriminating signal peptides from transmembrane regions. Nat. Met..

[25] Miller G.L. (1959). Use of Dinitrosalicylic Acid Reagent for Determination of Reducing Sugar. Anal. Chem..

